# MINocyclinE to Reduce inflammation and blood brain barrier leakage in small Vessel diseAse (MINERVA) trial study protocol

**DOI:** 10.1177/23969873221100338

**Published:** 2022-05-17

**Authors:** Robin B Brown, Daniel J Tozer, Laurence Loubière, Young T Hong, Tim D Fryer, Guy B Williams, Martin J Graves, Franklin I Aigbirhio, John T O’Brien, Hugh S Markus

**Affiliations:** 1 Department of Clinical Neurosciences, University of Cambridge, Cambridge, UK; 2 Wolfson Brain Imaging Centre, University of Cambridge, Cambridge, UK; 3 Department of Radiology, University of Cambridge, Cambridge, UK; 4 Department of Psychiatry, University of Cambridge, Cambridge, UK

**Keywords:** Stroke, small vessel disease, arteriosclerosis, neuroinflammation, bloodbrain barrier, clinical trial, experimental medicine

## Abstract

**Background:**

Cerebral small vessel disease (SVD) is a common cause of stroke and cognitive impairment. Recent data has implicated neuroinflammation and increased blood-brain barrier (BBB) permeability in its pathogenesis, but whether such processes are causal and can be therapeutically modified is uncertain. In a rodent model of SVD, minocycline was associated with reduced white matter lesions, inflammation and BBB permeability.

**Aims:**

To determine whether blood-brain barrier permeability (measured using dynamic contrast-enhanced MRI) and microglial activation (measured by positron emission tomography using the radioligand ^11^C-PK11195) can be modified in SVD.

**Design:**

Phase II randomised double blind, placebo-controlled trial of minocycline 100 mg twice daily for 3 months in 44 participants with moderate to severe SVD defined as a clinical lacunar stroke and confluent white matter hyperintensities.

**Outcomes:**

Primary outcome measures are volume and intensity of focal increases of blood-brain barrier permeability and microglial activation determined using PET-MRI imaging. Secondary outcome measures include inflammatory biomarkers in serum, and change in conventional MRI markers and cognitive performance over 1 year follow up.

**Discussion:**

The MINERVA trial aims to test whether minocycline can influence novel pathological processes thought to be involved in SVD progression, and will provide insights into whether central nervous system inflammation in SVD can be therapeutically modulated.

## Introduction

Cerebral small vessel disease (SVD) causes a quarter of strokes and is the commonest cause of vascular cognitive impairment. SVD refers to vasculopathy affecting small arteries and venules which show pathological changes including both focal microatheroma and more diffuse arterial deposits sometimes described as lipohyalinosis.^1^ Age, hypertension and diabetes are important risk factors for sporadic SVD. Characteristic MRI findings include small subcortical infarcts (lacunar infarcts), white matter hyperintensities (WMHs), cerebral microbleeds (CMBs) and enlarged perivascular spaces.^2^

A major obstacle to developing treatments for SVD is a gap in understanding of the pathogenesis and the lack of suitable surrogate disease markers. The conventional hypothesis is that the arteriopathy leads to a reduction in cerebral blood flow, and an impairment in cerebral autoregulation, which results in hypoperfusion. This hypothesis is supported by the finding that cerebral blood flow is reduced in the white matter of patients with SVD^3^ and that this reduction is seen not only in WMHs, but also in apparently normal appearing white matter.^4^

However, treatment of typical cardiovascular risk factors has proved largely unsuccessful in clinical trials,^5^ with the exception of intensive blood pressure treatment which reduced the progression of WMHs in hypertensive stroke-free individuals in the SPRINT-MIND study.^6^ Accordingly, other pathophysiological mechanisms have been investigated in an attempt to provide possible options for disease modifying treatment.

Two processes that have been implicated in the pathogenesis of SVD are inflammation, both systemic and central nervous system (CNS), and increased blood brain barrier (BBB) permeability. The role of systemic inflammation in cardiovascular disease is becoming better understood, and is thought to involve both vascular endothelium activation and cell-mediated components.^7^ Endothelial dysfunction and activation has been implicated in SVD and could mediate both the reduced flow and autoregulation.^8^ The endothelium in cerebral small arteries is abnormal on post mortem studies,^9^ and circulating endothelial markers are elevated in patients with SVD^10^; it has been further suggested that at least in some patients there may be a systemic endotheliopathy with additional abnormalities in systemic vessels.^11^ Systemic inflammatory markers were also found to predict SVD progression in longitudinal studies.^12^

In addition to evidence of peripheral inflammation in SVD, several studies have demonstrated inflammation in the CNS. Positron emission tomography (PET) imaging using the radioligand ^11^C-PK11195, which binds to activated microglia, has shown an association between whole brain inflammatory response and markers of SVD, including WMHs.^13^ Focal areas of increased binding (‘hotspots’) have also been demonstrated in patients with moderate to severe symptomatic SVD.^14^

Recently it has also been suggested that alterations in BBB permeability may be associated with the abnormalities in endothelial function and activation described above. Neuropathological studies show fibrinogen leakage around vessels consistent with BBB leakage,^15^ and a number of studies have reported evidence of low grade BBB permeability using dynamic contrast-enhanced MRI (DCE-MRI) in lacunar stroke^16,17^ and vascular cognitive impairment.^18^ More recent development of the technique allows maps of BBB permeability to be produced,^18^ and foci of increased BBB permeability have been shown in the white matter of patients with symptomatic SVD.^14^

A pathway linking hypoperfusion and hypoxia to BBB disruption and inflammation has been proposed based on studies in a rodent model of white matter ischaemia.^19^ This hypothesises that:

(1) Chronic hypertension causes vessel lumen narrowing and loss of cerebral autoregulation, leading to hypoperfusion and hypoxia in the vulnerable deep white matter
(2) Hypoxia then leads to production of HIF-1α, inducing an inflammatory response
(3) Matrix metalloproteinases (MMPs) are produced as part of this response, which disrupts the tight junctions and extracellular matrix of the vascular endothelium leading to opening of the BBB.

In the same rodent model minocycline administration was associated with a significant reduction in white matter damage and improved behavioural and survival outcomes.^20^ Minocycline is known to have anti-inflammatory properties within the brain, reducing the activation of microglia,^21^ and may be effective in stabilising the BBB.^22^

In the MINocyclinE to Reduce inflammation and blood brain barrier leakage in small Vessel diseAse (MINERVA) study, we are using a randomised controlled double-blind methodology to determine whether minocycline reduces neuroinflammation and BBB permeability, assessed using ^11^C-PK11195 PET and DCE-MRI respectively.

## Methods

### Aims and objectives

To determine whether minocycline reduces BBB permeability and microglial activation in patients with symptomatic SVD.

### Trial design

Phase II randomised, double-blind placebo-controlled trial of minocycline 100 mg twice daily for 3 months duration with surrogate outcome based on neuroimaging.

### Outcome measures

1. Primary co-endpoints:
  (a) Volume of ‘hotspots’ (see definition in Image Analysis below) of white matter BBB permeability measured on MRI
  (b) Volume of ‘hotspots’ of ^11^C-PK11195 binding in the white matter measured on PET
2. Secondary endpoints:
  (a) Mean BBB permeability and microglial activation of white matter measured using transfer constant and ^11^C-PK11195 binding potential respectively
  (b) Blood endothelial and inflammatory markers using the Olink proteomics platform (cardiovascular-III biomarker panel, www.olink.com/products-services/target/cardiovascular-iii-panel/)
  (c) Brain volume and WMH lesion volume change, and, change in markers of white matter microstructural integrity measured using diffusion tensor imaging (DTI) assessed on MRI at 12 months
  (d) Cognitive and behavioural metrics using a panel of neurospsychometric tests that we have previously optimised for use in this population^23^ based on assessments at baseline and 12 months (see Supplemental Table 1 for a list of tests used and domains assessed).

### Patient selection

**Figure 1.**
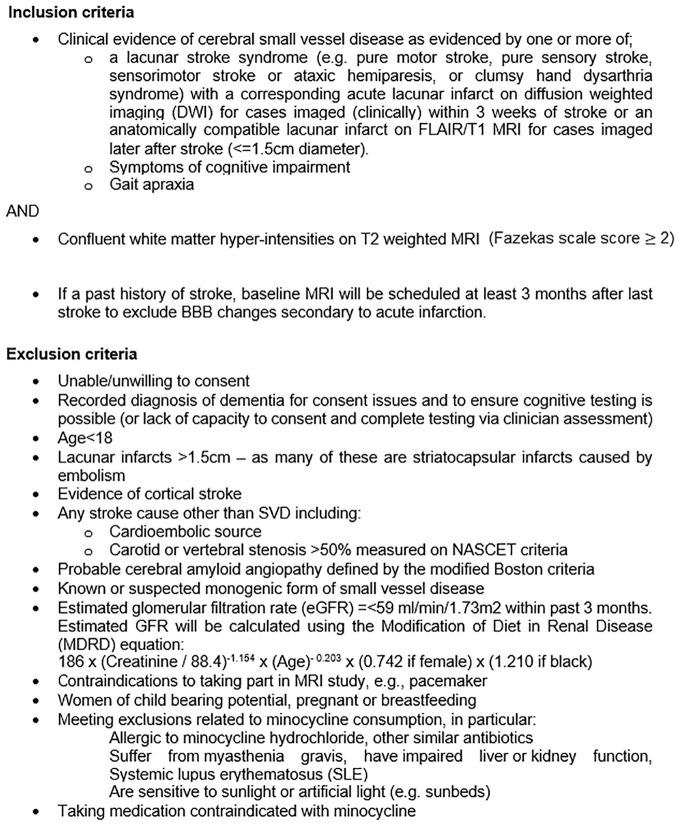
MINERVA inclusion and exclusion criteria.

Participants will be recruited from inpatient and outpatient stroke services at Cambridge University Hospitals NHS Foundation Trust and will have symptomatic small vessel disease as defined by a clinical syndrome compatible with SVD (lacunar stroke, cognitive impairment or gait apraxia) and at least moderately severe WMHs (Fazekas scale^24^ score ⩾2) Figure 1 shows the inclusion and exclusion criteria for the study.

We are not recruiting patients with cerebral amyloid angiopathy, which might have different pathological mechanisms and will exclude any participants with probable cerebral amyloid angiopathy defined using the modified Boston criteria.^25^ We are also excluding patients with known or suspected monogenic forms of SVD such as cerebral autosomal dominant arteriopathy with subcortical infarcts and leukoencephalopathy (CADASIL).

### Trial procedures and interventions

Patients are randomised to intervention or placebo in the ratio 1:1 with a random permuted block randomisation design (block size of 2/4). Randomisation is performed via a web-based system managed by Sealed Envelope Ltd (www.sealedenvelope.com). Participants in the intervention arm will take minocycline 100 mg orally twice daily; participants in the placebo arm will take a matching cellulose capsule. Participants and investigators are blind to treatment allocation.

**Figure 2.**
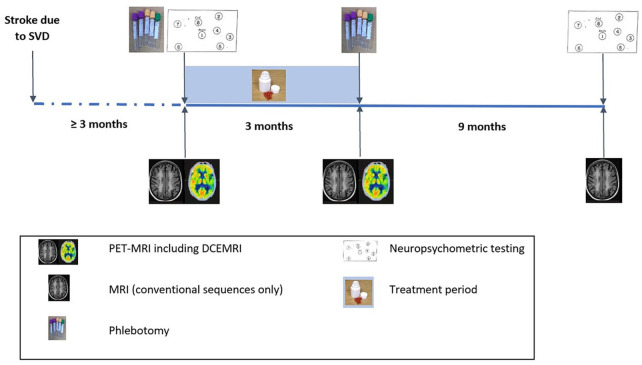
Structured trial timeline and procedures.

Participants undergo visits at baseline (for phlebotomy, neuropsychometry and PET-MRI imaging), 6 weeks (for clinical check-up), 3 months (for post-treatment data collection, phlebotomy and PET-MRI imaging) and again at 1 year for non-contrast MRI and repeat neuropsychometry. The trial design is summarised in Figure 2.

### Imaging acquisition

The trial neuroimaging protocol includes PET and MRI which is co-acquired on a 3T GE SIGNA PET-MRI scanner (GE Healthcare, Waukesha, WI) at the Wolfson Brain Imaging Centre in Cambridge, UK using sequences that we have previously optimised in a similar cohort.^14^ Baseline and 3 month (post-treatment) imaging includes:

1. PET data acquisition for 75 min following the injection of ^11^C-PK11195 (target injection activity 500MBq) produced at the Wolfson Brain Imaging Centre Radiopharmaceutical Unit
2. Simultaneous whole brain non-contrast MRI using a 32-channel head coil (Nova Medical) including T1- and T2-weighted images, FLAIR, DTI and susceptibility weighted images. Sequence details are given in Supplemental Table 2.
3. Dynamic T1 maps acquired using DCE-MRI in a sub-volume of the brain chosen to reflect characteristic damage due to SVD. A Gadolinium-based contrast agent in the form of gadoterate meglumine (Dotarem^®^) is injected at a sub-clinical dose of 0.025 mmol/kg. The dynamic T1 relaxation time is mapped prior to injection and is followed by eight cycles of post injection T1 mapping using an in-house developed pulse sequence that repeatedly acquires six 3D radiofrequency spoiled gradient echo images with different flip angles to calculate each T1 map.

Follow-up MRI only imaging is performed at 1 year and includes T1- and T2-weighted images, FLAIR, DTI and susceptibility-weighted images acquired on the same scanner.

### Image analysis

WMH lesions will be marked using Jim version 8.0 (http://xinapse.com/j-im-software/), a semi-automated program in which a region of interest is selected by the rater and voxels within this contour are selected. Pre- and post-treatment images will be marked slice by slice on a parallel split screen and displayed randomly in terms of order of acquisition to reduce the risk of bias with the rater blinded to image timepoint. T1 images will be processed to produce tissue probability maps for each tissue class after removal of the WMH mask. WMH and normal appearing white matter masks will then eroded by 3 mm to eliminate contamination from CSF or grey matter.

The T1 maps from the DCE-MRI will be calculated using the standard radiofrequency spoiled-gradient echo signal equation and used to estimate the gadolinium concentration in tissue using a Patlak graphical analysis to determine influx rate (K_i_) as a metric of permeability.^26^ As there is no artery in the field of view, we will use the superior sagittal sinus as an arterial input function, corrected by the factor (1–haematocrit), which is assumed to be representative of the arterial concentration of contrast agent.^18^ Voxels of increased BBB permeability (‘hotspots’) will be defined as those with a K_i_ greater than the 95th percentile of permeability derived from an existing cohort of stroke-free control participants.

List-mode PET data will be histogrammed into time bins and reconstructed using time-of-flight ordered subsets expectation-maximisation.^27^ Attenuation correction will include the use of a multi-subject atlas method^28^ and improvements to the MRI brain coil component. Image reconstruction will also correct for random coincidences, dead time, normalisation, scattered coincidences, radioactive decay, and sensitivity. SPM12 will be used to realign each dynamic image series which will be co-registered with the T1 MRI sequence using a mean realigned PET image.

**Figure 3.**
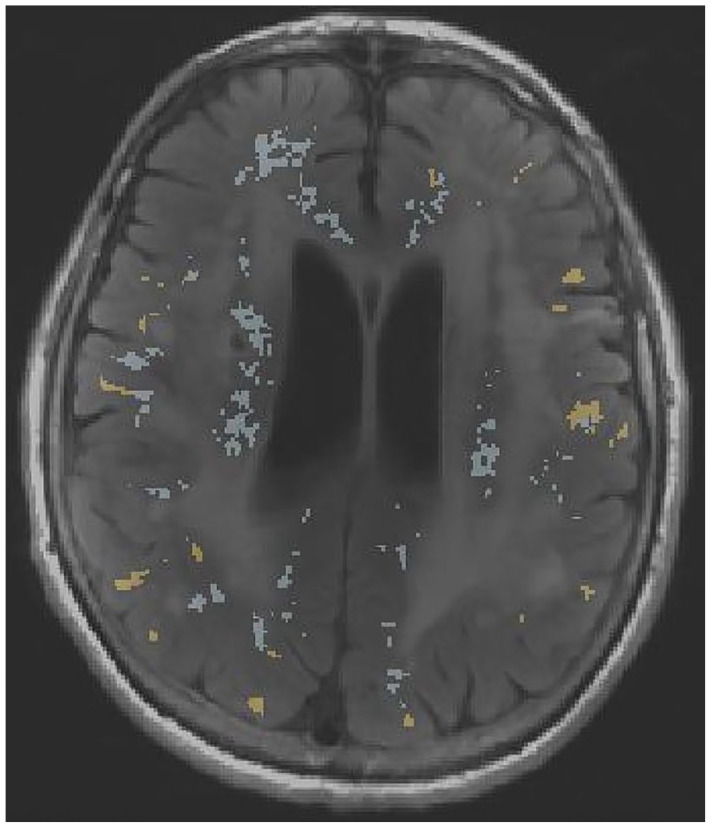
Sample FLAIR MRI image from a patient with sporadic SVD, with hotspots of microglial activation (yellow) and BBB permeability (blue) overlaid.

The specific binding of ^11^C-PK11195 will be estimated by determining the binding potential relative to a non-displaceable reference tissue (BP_ND_) using a basis function implementation of the simplified reference tissue model that incorporates correction for vascular binding.^29^ The white matter reference tissue input will be estimated with supervised cluster analysis^30^ using library data determined from healthy control participant ^11^C-PK11195 scans using on the same PET-MRI scanner. Binding hotpots will be defined as those above the 95th percentile of control participants as for the BBB permeability measurements above. Figure 3 shows an example of the maps of ^11^C-PK11195 binding and BBB permeability hotspots that can be produced using this technique.

DTI images will be analysed using FSL software (‘FDT’; FMRIB’s Diffusion Toolbox, http://fsl.fmrib.ox.ac.uk/fsl/fslwiki/FDT) to correct for eddy current effects and to create a binary brain mask in DTI space. Fractional anisotropy (FA) and mean diffusivity (MD) maps will be created from this data using the DTIFIT tool. Spurious cerebrospinal fluid voxels based on thresholds of MD values above 2.6 × 10^−4^ mm^2^ s^−1^ and FA >1 will be removed. For each participant, the FMRIB Linear Image Registration Tool will be used to register the FLAIR and B0 images. Tissue segments and WMH masks will then be transformed into DTI space and used to create tissue specific FA and MD histograms.

### Statistical analysis and power calculations

**Figure 4.**
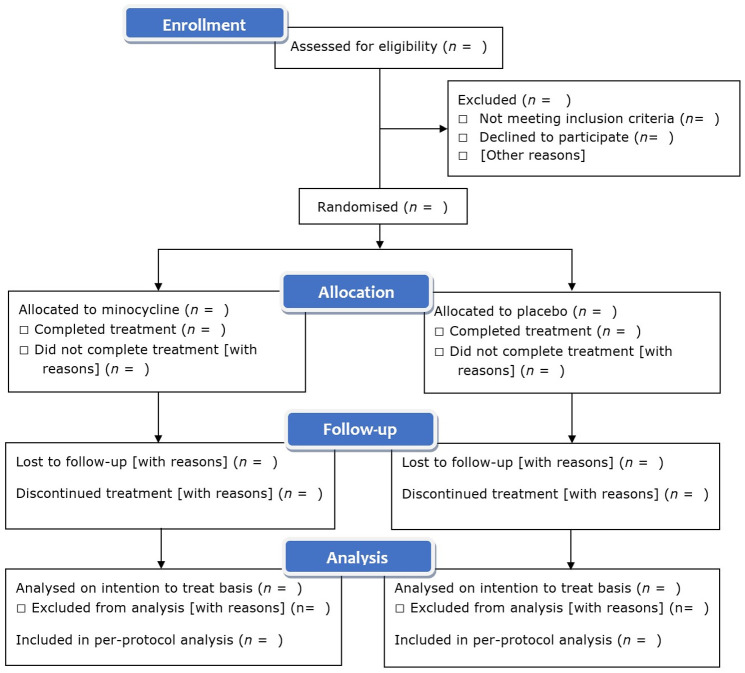
Template for summary of recruitment, treatment and analysis in trial CONSORT diagram.

Participant recruitment will be reported in a Consolidated Standards of Reporting Trials (CONSORT) diagram (template shown in Figure 4). Differences between the treatment and placebo groups will be tested using χ^2^ tests (categorical data) and one way ANOVA or Mann-Whitney *U* tests (for normal or non-normal continuous data) as appropriate.

Primary outcome analysis will be performed on an intention to treat basis, including all randomised participants. As our primary outcomes consider the treatment with minocycline as an experimental probe rather than a clinical endpoint, intention to treat analysis might bias the results towards the null hypothesis and so we will also perform a per-protocol analysis including only participants who complete the treatment course. Outcomes will be tested using standard regression models both unadjusted and adjusted for age, sex and demographic or clinical variables that are significantly different between groups.

Using the data from our observational study^14^ we calculated that in order to show a 20% reduction in ^11^C-PK11195 binding metrics with power of 80% and α = 0.05, we require 17 participants in each arm. To demonstrate a 20% reduction in BBB permeability with these constraints, we require 21 participants per arm. Our target sample size of 22 per arm encompasses these requirements.

### Safety and adverse event reporting

The radiation dose during PET imaging is approximately 2.6 mSv (the equivalent of 1 year of background environmental radiation). Participant information states that this confers a small additional risk of developing cancer, and patients consent explicitly to radiation exposure. 3T MRI does not have any adverse clinical effects, and as gadolinium contrast usage can lead to nephrogenic systemic fibrosis in participants with renal disease, only patients with an estimated glomerular filtration rate of 60 ml/min/1.73 m^2^ will be recruited.

Minocycline is a safe and well-tolerated medication but potential side effects include gastrointenstinal disturbance, dizziness and skin rashes/discolouration. Patients are provided with an alert card and emergency contact details for the study clinician in case of any possible side effects. Data will be collected on adverse reactions in keeping with the Summary of Product Characteristics for minocycline during the treatment period. Additional safety outcomes include recurrent stroke or other cardiovascular events during the treatment period and at 1 year, and change in neuropsychometric test performance.

### Data capture/data access

Data will be recorded electronically using an online research data management tool (REDCap) hosted at the University of Cambridge.^31^ REDCap (Research Electronic Data Capture) is a secure, web-based software platform designed to support data capture for research studies, providing validated data capture, audit trails for tracking data manipulation and export procedures, and procedures for importing data from external sources. After study completion, cleaning and database finalisation and our pre-specified analyses, anonymised data will be available for secondary uses on reasonable request.

### Ethical and regulatory approval

Approval for the MINERVA trial was granted by the East of England – Cambridge Central Research Ethics Committee (reference 18/EE/0237) and it has been classified as a non-CTIMP (clinical trial of investigational medical product) by the Medicines and Healthcare products Regulatory Authority. The use of ^11^C-PK11195 was approved by the UK Administration of Radioactive Substances Advisory Committee (ARSAC, Research ID 176; 19/09/2018). The study was registered prospectively on the International Clinical Trials Registry Portal (reference ISRCTN15483452).

## Discussion

The MINERVA study is testing the hypothesis that minocycline reduces measures of neuroinflammation and BBB permeability in a cohort of patients with symptomatic and moderate to severe SVD. It aims to test whether similar results can be demonstrated in patients with SVD to those reported in an rodent model of ischaemic white matter injury.^20^ Enrolment commenced in September 2019, with 34 participants enrolled by 01/03/2022, and is projected to finish midway through 2022.

Our primary outcomes will provide evidence as to whether this intervention can influence the inflammatory response in SVD based on ^11^C-PK11195 binding, and whether it can affect BBB permeability measured by DCE-MRI. If positive, it would imply that both processes can be altered in parallel, whereas if only one of the ^11^C-PK11195 PET or DCE-MRI outcomes are positive it would provide further evidence that these changes occur at different points in the disease process and should be targetted by different disease modifying strategies.

In addition to primary endpoints of microglial activation and BBB permeability, our study will also provide assessments of the effect of this intervention on radiological measurements of SVD severity and the activation of the systemic immune response using conventional MRI markers of SVD and immunophenotyping of peripheral blood respectively. Follow up after 1 year will allow us to assess any mid-term effects on brain structure/pathology and cognitive performance, though deteriorating cognition becomes evident over a longer timescale in SVD^32^ and longer term follow up would be required to accurately model the risk of incident stroke or cognitive impairment/dementia.

Potential limitations of our study include that the timecourse of the inflammatory response in SVD has not been fully characterised and in vivo evidence that microglial activation drives the progression of white matter damage is unclear. In addition, the PET radioligand we are using is known to have off-target binding^33^ and its receptor is expressed in multiple cell lines in addition to microglia^34^; our choice of reference tissue model aims to mitigate this by accounting for endothelial binding and thereby increasing the specificity for binding in brain parenchyma.

A final consideration is that the dose of minocycline that was effective in the rodent model^20^ may not translate to a human study. Minocycline has previously been tested unsuccessfully in neurodegenerative conditions such as Parkinson’s disease,^35^ and Alzheimer’s disease,^36^ but has not yet been assessed in SVD which has a different pathological mechanism to neurodegenerative causes of dementia.

Although both CNS inflammation and increased BBB permeability have been implicated in SVD, whether they play a causal role and whether they can be therapeutically modulated in man is uncertain. The MINERVA study will provide novel information in this area, and if positive will inform phase 3 trials of immunomodulatory therapy in SVD.

## Supplemental Material

sj-docx-1-eso-10.1177_23969873221100338 – Supplemental material for MINocyclinE to Reduce inflammation and blood brain barrier leakage in small Vessel diseAse (MINERVA) trial study protocol Click here for additional data file. Supplemental material, sj-docx-1-eso-10.1177_23969873221100338 for MINocyclinE to Reduce inflammation and blood brain barrier leakage in small Vessel diseAse (MINERVA) trial study protocol by Robin B Brown, Daniel J Tozer, Laurence Loubière, Young T Hong, Tim D Fryer, Guy B Williams, Martin J Graves, Franklin I Aigbirhio, John T O’Brien and Hugh S Markus in European Stroke Journal
