# Supplementary material for: MINocyclinE to Reduce inflammation and blood brain barrier leakage in small Vessel diseAse (MINERVA) trial study protocol
Source: Eur Stroke J. 2022 May 17;7(3):323–30. doi: 10.1177/23969873221100338 (PMC9445404; doi:10.1177/23969873221100338)
Supplement: sj-docx-1-eso-10.1177_23969873221100338 – Supplemental material for MINocyclinE to Reduce inflammation and blood brain barrier leakage in small Vessel diseAse (MINERVA) trial study protocol [file sj-docx-1-eso-10.1177_23969873221100338.docx]

**SUPPLEMENTARY MATERIAL.**

Supplementary Table 1. Cognitive domains assessed and neuropsychometric tests used at baseline and one year.

| **Neuropsychometric domain** | **Assessments used** |
| --- | --- |
| Working memory | Digit span |
| Episodic (long term) memory | Logical memory I & II and visual reproduction I & II from the Wechsler Memory Scale-IV (www.pearsonclinical.co.uk) |
| Processing speed | Digit symbol substitution, Brain Injury Rehabilitation Trust Memory and Information Processing Battery (www.thedtgroup.org/research/bmipb), and the grooved pegboard task (https://www.advys.be/docs/) |
| Executive function | Trail-making test (part B, Reitan, R. M., & Wolfson, D. (1985). *The Halstead–Reitan Neuropsycholgical Test Battery: Therapy and clinical interpretation*. Tucson, AZ: Neuropsychological Press), single letter verbal fluency, and the Wisconsin card sort test (Grant, D. A., Berg, E. (1948). A behavioral analysis of degree of reinforcement and ease of shifting to new responses in a Weigl-type card-sorting problem. Journal of Experimental Psychology, 38, 404-411) |
| Mood assessment (apathy and depression) | Geriatric Depression Scale (http://www.stanford.edu/~yesavage/GDS.html) |
| Fatigue severity assessment | Fatigue severity scale (Krupp, L. B., LaRocca, N. G., Muir-Nash, J., & Steinberg, A. D. (1989). The fatigue severity scale. Application to patients with multiple sclerosis and systemic lupus erythematosus. *Archives of Neurology, 46*, 1121–1123) |

Supplementary Table 2. MRI sequence acquisition details.

| **Sequence** | **Acquisition parameters** |
| --- | --- |
| T_1_-weighted | Axial 3D fast-spoiled gradient echo sequence (BRAVO), flip angle = 12°, inversion time = 450ms, field of view =28 mm, slice thickness = 1mm, number of slices =192, reconstructed matrix size = 512 × 512 |
| T_2_-weighted | Axial T_2_ fast-spoiled gradient echo sequence angled anterior commissure-posterior commissure (AC-PC), flip angle = 111°, TE = 85 ms, TR = 6000ms, field of view = 22mm, slice thickness = 5 mm, number of slices = 31, reconstructed matrix size = 1024 × 1024 |
| FLAIR | Axial T_2_ FLAIR, angled AC-PC, flip angle = 160°, TR = 8800ms, TE = 120ms, TI = 2445ms, field of view = 22mm, slice thickness = 5 mm, number of slices = 28, reconstructed matrix size = 256 × 256 |
| Susceptibility-weighted | Axial susceptibility weighted imaging, flip angle = 17°, repetition time = 40.6ms, echo time = 24.2ms, field of view = 22 mm, slice thickness = 2mm, number of slices = 70, reconstructed matrix size = 256 × 256 |
| DTI | Axial DTI, angled anterior commissure-posterior commisure with the diffusion gradients applied in 63 directions with a b-value = 1000s/mm^2^, TE = minimum, TR = 15763ms, field of view = 19.2mm, slice thickness = 2 mm, number of slices = 65-70 depending on slice angulation reconstructed matrix size = 256 × 256 |
| DCE-MRI | 3D radiofrequency spoiled gradient echo, TR = 6.3ms, TE = 1.784ms, number of slices = 16, reconstructed matrix size = 256 × 256, final resolution = 0.94 × 0.94 × 3mm, flip angles = 2°,5°,12°,17°,22°,27°, temporal resolution = 15 seconds per flip angle with interphase interval 15 seconds (eight cycles) |
